# Factors Associated with COVID-19 Vaccine Hesitancy in South Central Appalachia

**DOI:** 10.13023/jah.0503.06

**Published:** 2023-12-01

**Authors:** Florence M. Weierbach, Rebecca Adkins Fletcher, Ingrid E. Luffman, Cynthia Meyer, Janet M. Keener, Manik Ahuja, Hadii M. Mamudu

**Affiliations:** East Tennesee State University; East Tennesee State University; East Tennessee State University; East Tennessee State University

**Keywords:** Appalachia, COVID-19, health behaviors, spatial analysis, vaccine hesitancy

## Abstract

**Introduction:**

The newly emergent COVID-19 virus reached pandemic levels in March 2020. By the middle of August 2020, there were over 1 million deaths attributed to COVID-19 in the U.S., with those in rural areas outpacing urban counterparts. Prior to emergency approval of the Pfizer, Moderna and Johnson & Johnson vaccine formulations, mitigation efforts addressing individual behavior were challenging. However, even with the entrance of these three new vaccines, herd immunity was not achieved in rural areas, as vaccine uptake remained low there. Although there has since been an abundance of COVID-19-related research addressing health literacy, vaccine hesitancy and overall medical mistrust, few of these studies focus on Appalachia.

**Purpose:**

This study identifies barriers and facilitators to adherence with COVID-19 mitigation, focusing specifically on vaccine hesitancy in South Central Appalachia.

**Methods:**

A secondary data study was conducted with a subset of Appalachian residents from the COVID-19 Public Health survey. Participants were grouped by county using ARC economic county designations for analysis. The dependent variable, vaccine hesitancy, was explored in relation to five categories of independent variable: (1) demographics (with four conceptual areas); (2) belief; (3) action; (4) medical mistrust; and (5) health literacy.

**Results:**

Findings indicate vaccine hesitancy attributes include beliefs addressing COVID-19 threat, overstatement of severity of illness, risk of vaccines, vaccine safety information not present from manufacturer, and independent decision to vaccinate. Findings from this study are comparable to HPV vaccine studies in Appalachia.

**Implications:**

As interventions are developed for Appalachia, it is paramount to focus vaccine administration at the individual and population level.

## INTRODUCTION

In March 2020, the newly emergent COVID-19 virus reached pandemic levels.^1^ As of mid-summer 2023, over 1.1 million deaths have been attributed to the virus in the U.S.^2^ Collectively, nonmetropolitan area COVID-19 death rates range from 1.11 to 7.99 per 100,000, compared to metropolitan death rates at 0.46 to 8.05 per 100,000.^2^ After the initial peak of deaths from March to July 2020, nonmetropolitan deaths increased, matched, and at times exceeded metropolitan rates.^2^ Nonmetropolitan rural areas (less than 2,500 residents) recorded higher COVID-19 death rates than other areas. These deaths have been attributed to increased vulnerability to serious infection due to an older population, greater incidence of underlying medical conditions, lack of health insurance, and greater distance to an intensive care hospital.^3^ Early approval of three COVID-19 vaccines was thought to be the panacea to addressing the pandemic; with the aid of vaccines, researchers believed herd immunity for COVID-19 was a viable public protection strategy and could be established if 75–90% of the population was vaccinated.^4^,^5^ However, according to the U.S. Centers for Disease Control and Prevention (CDC),^6^ overall COVID-19 vaccination coverage for adults was lower in rural counties (38.9%) than in urban counties (45.7%). Vaccine hesitancy has been identified as a barrier for vaccination, and several studies have examined it in rural populations.^4^,^7^,^8^

A broad definition of vaccine hesitancy includes the concepts of complacency, confidence and convenience,^9^ while a narrow definition—“the reluctance or unwillingness to be vaccinated or have ones children vaccinated against a disease, even if proven safe and effective”^10^—allows alignment of individual behaviors to one of these three concepts. Specific barriers in rural areas have been attributed to transportation and availability, medical mistrust, and health literacy,^4^,^7^,^8^ which align with the concepts of confidence and convenience. Transportation to available vaccination sites in rural communities may be attributed to the concept of convenience. Vaccination for rural residents requires individuals to identify vaccination sites, times the vaccine is available, and transportation to the site.

A separate barrier aligned with confidence is mistrust, which includes medical mistrust but may also extend to fear and lack of trust in the government. Medical mistrust may prevent individuals from seeking, receiving, and following treatment recommendations; vaccine hesitancy has been identified as one of its consequences.^11^ For example, rates of COVID-19 vaccine hesitancy in Arkansas were found to be higher among black residents, due in part to distrust of the medical system associated with racism and discrimination.^12^ Other factors that contribute to COVID-19 vaccine mistrust in the U.S. include the speed of vaccine development and emergency authorization use, the politicization of vaccines, mistrust of science and doctors, mistrust of government, and unethical research practices involving under-represented groups^5^,^7^.

An additional barrier aligned with confidence is vaccine hesitancy. In rural areas, this COVID-19 vaccine hesitancy has been tied to health literacy, though the exact association between the two remains ambiguous.^12^,^13^ Rural communities tend to have lower health literacy rates,^14^ which may impact vaccine uptake and other mitigation efforts due to misunderstanding or misinformation on the vaccine. Kricorian et al.^13^ found that study participants who believed the vaccine was unsafe expressed vaccine hesitancy and were less likely to get information from trusted scientific sources.^12^ However, a study in progress using the Public Health COVID-19 survey among patients with chronic diseases and community members residing in southern states did not find any significant association between health literacy and COVID-19 vaccine hesitancy. Limited rural transportation options in combination with medical mistrust and health literacy are challenging to address, and efforts are further complicated due to rural residents’ past experiences with government and health providers.^4^,^7^,^8^

While there are emerging studies about COVID-19 vaccine hesitancy in rural communities,^13^ research is limited in Appalachian counties. Thus, the present study identifies barriers and facilitators to the adherence to COVID-19 mitigation measures in South Central Appalachia, with focus on vaccine hesitancy among a subset of residents. This analysis focuses on identification and model building and addresses COVID-19 vaccine uptake and hesitancy in this population, which is at high risk for COVID-19-induced hospitalization and fatalities.

## METHODS

### Participants

This study comprises a subset of adults who responded to the COVID-19 Public Health Survey conducted by the Center for Cardiovascular Risks Research (CCRR) at East Tennessee State University (ETSU). This survey sought to understand and identify barriers and facilitators to adherence to COVID-19 mitigation measures, testing, and vaccine uptake and hesitancy among a population at high risk for COVID-19-induced hospitalizations and fatalities. This purpose guided a literature review conducted by CCRR clinicians and research hers in the course of survey development.

Upon IRB approval, the secure REDCap survey was administered online from February to June 2021. Recruitment was targeted to individuals with chronic health conditions, community stakeholders (local government and business owners), and health care providers who were involved in the CCRR research initiative at ETSU. Additional recruitment strategies included survey invitations present on the CCRR website and distribution of invites by CCRR associate individual networks via social media, targeted emails, and regional conference attendance. Further recruitment included a snowball approach in which survey respondents were asked to share the survey link with their family members, friends and colleagues.

**Figure 1:**
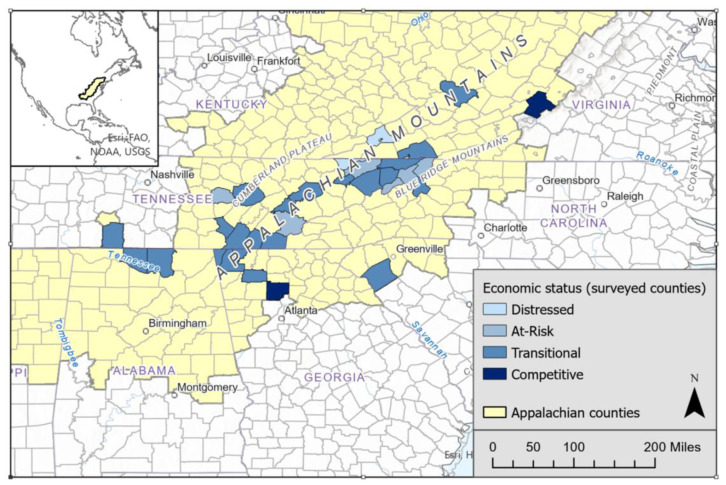
Economic status of Appalachian counties with survey responses

A total of 417 individuals participated in the online survey. The sample for this secondary analysis includes 181 participants from 32 counties in 6 Appalachian states. Counties with only one or two respondents were excluded, and the original 417 participants were reduced to 181 for the county-level analysis (Fig. 1). Counties were categorized using ARC economic designations: distressed, at risk, transitional, and competitive.^15^ Counties with a stronger economic designation have both rural and urban areas, while counties with weaker designation are rural counties.^15^

### Variables and Measures

#### Dependent Variable

**Figure 2:**
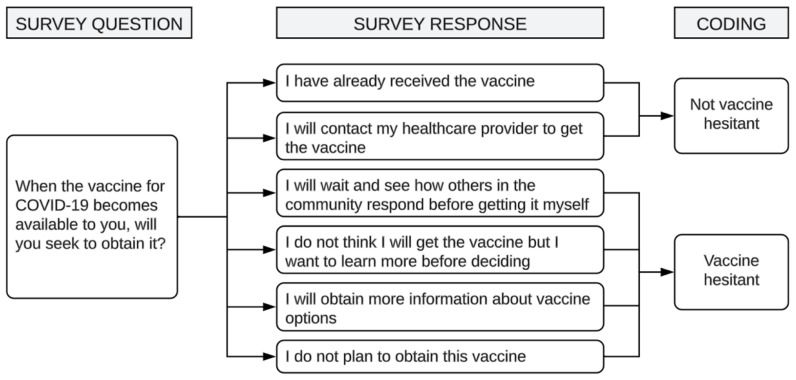
Decision tree survey question and responses addressing vaccine hesitancy

The main outcome variable was COVID-19 vaccine hesitancy. Survey respondents were categorized as vaccine hesitant by evaluating their responses to a standard and validated question (Fig. 2). A new dummy variable was created with a value of ‘1’ if the respondent was classed as vaccine hesitant and ‘0’ if not.

#### Independent Variables

Difference in vaccine hesitancy by county economic status was examined using a Kruskal–Wallis test. Independent variables of interest were then identified from the COVID-19 Public Health survey as either demographics or falling within one of four conceptual areas: belief, action, medical mistrust, or health literacy. These independent variables were recoded using dummy variables where necessary to enable statistical analyses (Table 1). Spearman’s correlation coefficient was calculated in IBM SPSS version 27, and variables with a significant correlation to the dependent variable were selected for inclusion in a logistic regression model. Logistic regression models for vaccine hesitancy and selected independent variables were assessed for overall percentage correct using a classification table with a cut value of 0.24. The cut value was selected to maximize specificity and sensitivity. Because the aim of modeling was to identify factors associated with vaccine hesitancy, not build a predictive model, the full dataset was used, and no records were reserved for model validation.

## RESULTS

Survey respondents had an average age of 48.8 years (range: 21–83 years) and were primarily female (81%) and white (94%). Over half of respondents held a bachelors, associates, or vocational trade school degree (52%), and a further 30% held a graduate degree. The remaining 13% of respondents had a high school education or less. Annual household income was balanced among respondents, with 30% earning less than $50k, 43% earning 50–100k, and 27% earning greater than $100k. Most respondents’ health insurance was supplied through their employer (62%), with 18% on Medicare, 6% having private insurance, 6% insured through the marketplace, and the remaining on Medicaid, VA/TriCare, or uninsured. Nearly half of respondents self-identified as having a chronic medical condition (45%), and 17% were licensed healthcare providers.

Individual vaccine hesitancy responses were aggregated to the county level, and county vaccine hesitancy was not statistically related to county economic classification. This is likely due to the way in which survey respondents were distributed between the four economic status categories; 162 of 181 respondents were in counties classed as “transitional.”

### Correlation between vaccine hesitancy and independent variables

Of the variables listed in Table 1, Spearman’s correlation results show several independent variables highly correlated with vaccine hesitancy: belief that COVID-19 is not a severe threat to the respondent (*r* = 0.405, *p* < .01); belief that severity is overstated (*r* = 0.470, *p* < .01); a personal choice to avoid vaccines (r = 0.454, *p* < .01); concerns about risks of vaccination (*r* = 0.524, *p* < .01); and belief that adequate information about safety of vaccine was not provided by manufacturers (*r* = 0.569, *p* < .01).

A logistic regression model on the dependent variable, *vaccine hesitant*, was developed with all significantly correlated covariates (Table 1). Using a cut value of 0.24, the model correctly classified 85.2% of respondents by vaccine hesitancy status (Table 2), with 18 respondents incorrectly classified as vaccine hesitant (false positives) and two respondents incorrectly classified as not vaccine hesitant (false negatives). The Nagelkerke R-square value was 0.641, indicating a good model. Due to incomplete surveys, only 135 participant surveys were used in model development.

Independent variables associated with vaccine hesitancy retained in the logistic regression model (Table 2) show that respondents who felt that COVID-19 was not a risk to themselves were 45 times more likely to be vaccine hesitant than those who felt COVID-19 presented a personal risk. Further, respondents with concerns about the safety of the vaccine (*Concerns*) were over 48 times more likely to be vaccine hesitant than those without concerns about vaccine safety.

## DISCUSSION

In a thorough examination of the variables and COVID literature, five broad areas were assigned to each of the survey items: demographics, actions, beliefs, mistrust, and health literacy. Each survey area was identified and assigned to the individual survey item (Table 1). Three of the five areas overlap and are highly correlated with each other. The overlapping correlated variables align with action, belief, and medical mistrust. The two covariates retained in the final vaccine hesitancy model address an individual’s belief and concern. With so few variables aligning with health literacy, it is difficult to ascertain its contribution to the overall question addressing vaccine hesitancy. From the correlated variables, belief is statistically significant, while action and medical mistrust are not. The significant covariates retained in the final model focus on belief and concern, and medical mistrust covariates were not retained in the model. This provides justification for concern to be identified as a separate concept or as an antecedent to medical mistrust.

The two covariates in the final model represent belief and medical mistrust, while the dependent variable, *vaccine hesitancy*, represents action. These findings suggest that actions are associated with beliefs and trust. It is important to note that the survey question *trust in healthcare providers* was not significantly correlated with vaccine hesitancy or other survey questions assigned to the areas of medical mistrust or health literacy.

This survey identifies several attributes of vaccine hesitancy (covariates with high correlation) among survey participants in South Central Appalachian counties. These include: (1) belief that COVID-19 is not a severe threat to the respondent; (2) a personal choice to avoid vaccines; (3) belief that severity [of COVID-19] is overstated; (4) concerns about risks of vaccination; and (5) belief that adequate information about safety of the vaccine was not provided by manufacturers. These five attributes align with vaccine hesitancy concepts of complacency and confidence. The first two attributes addressing severity of COVID-19 align with complacency, while the three remaining align with confidence.^9^ All of the attributes signal valuations of risk by survey participants that differ from public health models, with the perceived risk from COVID-19 and severity of disease less than the perceived risk from the vaccine. This is supported by the logistic regression model, where participants with a reduced perception of personal COVID-19 risk were 45 times more likely to be vaccine hesitant. Similarly, respondents concerned with the safety of the vaccine were 48 times more likely to be vaccine hesitant. Put simply, the survey results point to increased vaccine hesitancy among Appalachian survey respondents when they are more concerned (perceive greater personal risk) about the safety of the vaccine (medical mistrust) than about their risks from COVID-19 (belief/personal risk assessment).

## IMPLICATIONS

Vaccine uptake hesitancy is a historic aspect of preventive public health measures, and it is uneven over time and across U.S. regions. Appalachia offers a recent example of this regarding vaccine hesitancy associated with the HPV (human papillomavirus) vaccine, a vaccine first available in 2006 and developed to protect against sexually transmitted infections (STIs) that can lead to cervical and other cancers. Appalachian Kentucky has long been recognized for high rates of cervical cancer (incidence, morbidity, and mortality).^16^ However, Appalachian Kentuckians, like many other rural U.S. residents, have low HPV vaccine uptake rates compared with U.S. urban residents and those in other regions.^15^

Lessons from HPV vaccine uptake in Appalachia offer important and correlating lessons with COVID-19 vaccine hesitancy in Appalachia. First, HPV and COVID- 19 vaccine uptake rates are place-based and vary across the Appalachian subregions; care should be taken to avoid homogenizing descriptions of rural and Appalachian residents. It is necessary to consider rural differences and dynamics in “culture, economic hardship, community identity, and values” to effectively assess barriers and facilitators for HPV vaccine uptake.^16^,^17^ This also holds true for COVID-19 vaccine hesitancy and should inform future research aimed at understanding the factors apprising perceptions of safety of the COVID-19 vaccine (medical mistrust) and factors informing personal perceptions (beliefs) of risk from COVID-19. Second, the findings of this survey regarding the COVID-19 vaccine are in line with health belief models of HPV vaccine uptake, where vaccine hesitancy is higher among those perceiving the safety of the vaccine as a greater threat than its correlate disease.^18^,^19^

Limitations are present with this study. The sample size is limited by the number of Appalachian states and counties, with the inability to provide a comprehensive look at ARC county-level designation due to the geographic distribution of respondents. Additional limitations include recruitment and subsequent sample size, which does not reflect the demographics of rural or urban Appalachia. The online platform for the survey limits participants to those who have internet access, which is not readily available everywhere in rural Appalachia. Furthermore, the five survey areas—demographics, actions, beliefs, mistrust, and health literacy—were identified by clinical experts and have not been validated with a factor analysis of the COVID19 Public Health Survey.

Despite these limitations, findings demonstrate the need for culturally informed, regionally sensitive education and interventions to support vaccine uptake. In Appalachia, as elsewhere, it is imperative that interventions for vaccine administration address belief and concern at both the individual and population level.

**SUMMARY BOX**

**What is already known about this topic?**

Research centering on other vaccines, such as HPV vaccination studies, address the perception of vaccine threat as higher than that of contracting the virus. Since the emergence of COVID-19, there has been an abundance of research into health literacy, vaccine hesitancy, and overall medical mistrust across the pandemic. However, there is a dearth of research exploring these concepts within Appalachia.

**What is added by this report?**

The findings of this analysis, which relies on county-level economic classifications, indicate vaccine beliefs about safety and adequacy of vaccine manufacturer information which outweigh perceptions of the severity/threat of COVID-19 to individuals.

**What are the implications for future research?**

The findings demonstrate a need to conduct vaccine intervention research at the population level to address cultural beliefs and concerns, particularly medical mistrust. Additional insights could emerge from factor analysis of the ETSU CCRR’s COVID 19 Public Health Survey.

## Figures and Tables

**Table 1:** COVID-19 Public Health Survey question and Spearman correlation with vaccine hesitancy

Description: Survey question and responses	Correlation	Grouping of Variables

Highest level of education received	−0.174*	Demographic
		Belief
COVID-19 is a risk to me	0.405†	

COVID-19 is a risk to my community	0.334†	

COVID-19 is a risk to the nation	0.334†	

Nervous system disorder	0.221†	Demographic

Severity of disease is overstated	0.470†	Belief

Respondent follows prevention measures:		Action
1: Wear mask in public	1: −0.186*	
2: Physical distancing	2: −0.226†	
5: Limit social gatherings	5: −0.240†	

Respondent not routinely vaccinated for flu	−0.396†	Action

Reasons for not receiving vaccinations:		Belief
2: Religious practices	2: 0.454†	
5: Personal choice	5: 0.454†	

Concern for vaccine safety	0.524†	Medical Mistrust

Believes other ways to prevent transmission other than vaccine	0.235†	Belief

Pharmaceutical manufacturers do not provide adequate information on safety/efficacy of vaccine	0.569†	Medical Mistrust

Where vaccine received:		Action
4: Health department	4: −0.284†	
5: Hospital	5: −0.162*	

Factors contributing to vaccine avoidance:		
1: Preexisting medical condition	1: −0.162*	1: Medical Mistrust
2: Spiritual beliefs	2: 0.158*	2: Belief
3: Personal beliefs	3: 0.224†	3: Belief
5: Concerns about safety	5: 0.357†	5: Health Literacy
6: Concerns about freedom of choice	6: 0.275†	6: Health Literacy
7: Concerns about use of health data	7: 0.198*	7: Health Literacy

NOTES: Only significantly correlated covariates are displayed.

* Significant at *p* = .05

† Significant at *p* = .01

**Table 2:** Logistic regression results: classification table and retained covariates

Observed	Predicted			% Correctly classified
	Not vaccine hesitant	Vaccine hesitant		
Not vaccine hesitant	101	18		84.9

Vaccine hesitant	2	14		87.5

	**Overall % correctly classified**			**85.2**

**Covariate**	**Description**	**Standard Error**	***p*** **-value**	**Odds**

Belief1_n	COVID-19 not a risk to me	1.373	0.006	45.034

Concerns	Concerned about vaccine safety	1.373	0.005	48.479
